# Structural Probes in Quadruplex Nucleic Acid Structure Determination by NMR

**DOI:** 10.3390/molecules171113073

**Published:** 2012-11-05

**Authors:** Andreas Ioannis Karsisiotis, Mateus Webba da Silva

**Affiliations:** Biomedical Sciences Research Institute, University of Ulster, Cromore Road, Coleraine, Co. Londonderry BT52 1SA, UK

**Keywords:** chemical modifications, 8-bromoguanosine, quadruplex, 2,6-diaminopurine, LNA, inosine, 8-*O*-methylguanine, 8-methylguanine, 5-bromouracil, riboguanosine

## Abstract

Traditionally, isotope-labelled DNA and RNA have been fundamental to nucleic acid structural studies by NMR. Four-stranded nucleic acid architectures studies increasingly benefit from a plethora of nucleotide conjugates for resonance assignments, the identification of hydrogen bond alignments, and improving the population of preferred species within equilibria. In this paper, we review their use for these purposes. Most importantly we identify reasons for the failure of some modifications to result in quadruplex formation.

## 1. Introduction

**Figure 1:**
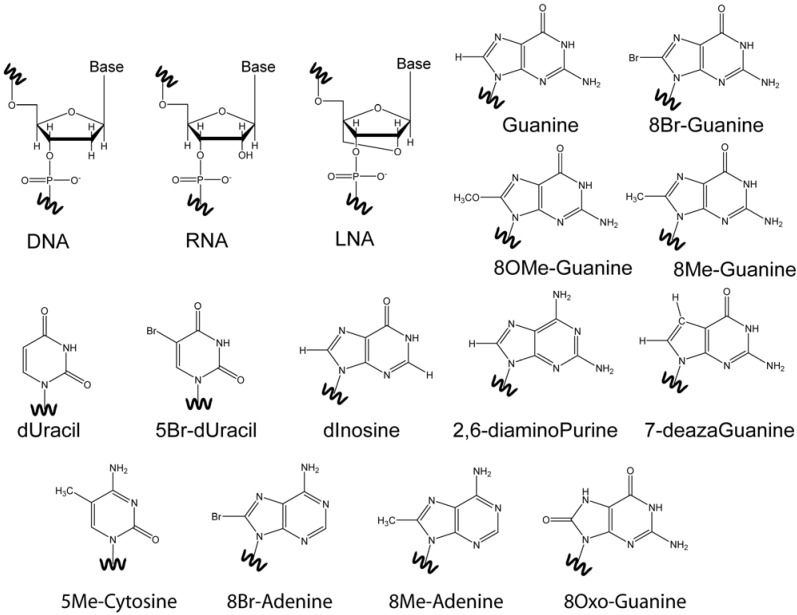
Chemically modified nucleobases utilized in solution NMR structure determinations.

DNA usually adopts a structure in which its strands are connected through hydrogen bond alignments of G:C and A:T into a topology with two grooves. Guanine-rich nucleic acids may form nucleic acid architectures that have four bases participating in pseudo-planar hydrogen bond alignments that define topologies that have four grooves. These are known as DNA quadruplexes. These architectures are of interest due to their biological functions, as well as utility as therapeutics, and materials. They add to the chemical diversity of structures with useful functions. Solution NMR spectroscopy has played a crucial role in the initial developments in the study of quadruplex DNA. The motivating premises for the study of quadruplexes followed a train of events that were predicated on structure. Some of the initial general questions were about the hydrogen bonding alignments feasible, the bases that can participate, how are they stabilized, *etc*. The uses for these architectures were initially speculative; and remained so for a long period of time. As with any chemical entity, to develop understanding on their use, or their biological function, we have always had the need to understand structure. Solution NMR structural studies are ever more useful in this context. There are a number of reviews that focus on the methodologies that have been used [1], and on how specific structural characteristics can be observed in quadruplexes by solution NMR [2]. The modified nucleosides shown in Figure 1 have been used as probes for structure determination, but also in their own right as part of quadruplex structure for varied reasons. A number of reviews have been written on modified G-quadruplex nucleic acids [3,4], but none specific to structure determination. Here we review modified nucleic acids that can be used to aid in the determination of solution quadruplex structure by NMR and how they influence the folding of the topologies examined.

## 2. The Structure of Quadruplexes

**Figure 2:**
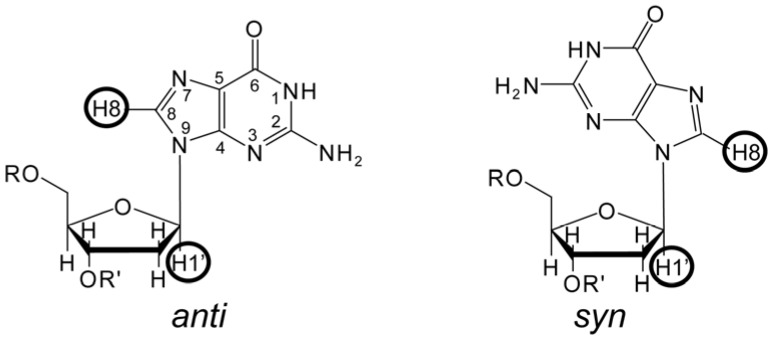
Chemical structures of the *syn* and *anti* glycosidic bond angles of guanosine.

Quadruplexes are thus composed of four hydrogen bond aligned bases, denominated tetrads, that stack onto each other to form a well-defined nucleic acid stem. It has four grooves whose width is defined by the angular relationship defining each of the bases to their sugar pucker: they can be narrow, medium, or wide. This relationship is best defined by the glycosidic bond angle (GBA) of the bases that make up the quadruplex stem—Figure 2. We have previously defined the relationships between structural parameters of these architectures based on a geometric formalism defined by this relationship [5]. The primary requirement for formation of a quadruplex stem is thus that a minimum of two stacked tetrads with the same groove widths stack onto each other. In a reductionistic sense there can be two states for the GBA: one in which the H1' of the sugar is *trans* (*anti*) with respect to the H8 of the base, and the other in which their relationship is *cys* (*syn*). The position of specific GBAs within the quadruplex stem defines, and is defined by, the topology of the architecture. Other tetrads, triads and mismatches can stack onto a quadruplex stem. Furthermore, tetrads may include bases other than guanosines.

## 3. The Identification of NMR Signals

The process of determining solution structures of quadruplexes by NMR starts of with a solution containing a DNA or RNA molecule of interest, with known sequence of bases. The initial steps involve the identification of ^1^H signals corresponding to the sequence of residues in isotopic natural abundance samples: the sequence specific assignment. Initially the distances between the H8 proton of the base and the H1' of the sugar of a nucleoside are fundamental for identifying sequence through NOE-based experiments. The identification of other proton signals within the nucleoside can allow for this sequential identification. The sequence of nucleosides can also be followed through scalar coupling NMR experiments that correlate protons of two sequential sugar puckers. However, for a number of reasons such as difficulties in identifying sequential steps involving G(*syn*) residues, major overlap of sugar proton signals, and others that reside outside the scope of this review, the sequential assignment of oligonucleosides may not possible. These issues can be addressed by the use of modified oligonucleosides.

## 4. Base Modifications

Base substitutions are essential tools in the identification of NMR signals, but also in imparting biological or thermodynamic stability to particular topologies. Their use is now widespread due to the development of solid phase synthesis methods. One of the most commonly used base modifications is the substitution of the H8 of guanosine by Br-, methyl-, *O*-methyl-, amino-, and oxo-substituents. Its origins reside in the need to define the sequence specific assignment of residues in a DNA molecule. This substitution would result in the disappearance of NOE cross-peaks due to the H8 proton. Instinctively, it is expected that the substitution of H8 is associated with an increase of steric effects due to the size of the substituent. Thus all of these substituents result in locking, or inducing the relationship between base and sugar to a G(*syn*). Most of the substitution studies were performed with the bromo substituent. Various studies have shown that it is feasible to identify the G(*syn*) guanosines of a topology through systematic substitution of the guanosine bases in its sequence. However, the substitution of specific G(*syn*) bases did not result in folded topologies. Here we provide a plausible explanation.

### 4.1. 8-Br-Guanosine Substitutions Prevent Formation of Narrow Grooves

Modified bases usually do not contribute to the stability of a quadruplex, with modifications at the 8 position being a notable exception. C8 modifications do not disrupt the Hoogsteen edge hydrogen bonding within the G-tetrad and positioned in the grooves of the quadruplex structure. The position of the C8 modified Guanine within the quadruplex stem plays a role on formation of quadruplexes, as well as its stability [6].

**Figure 3:**
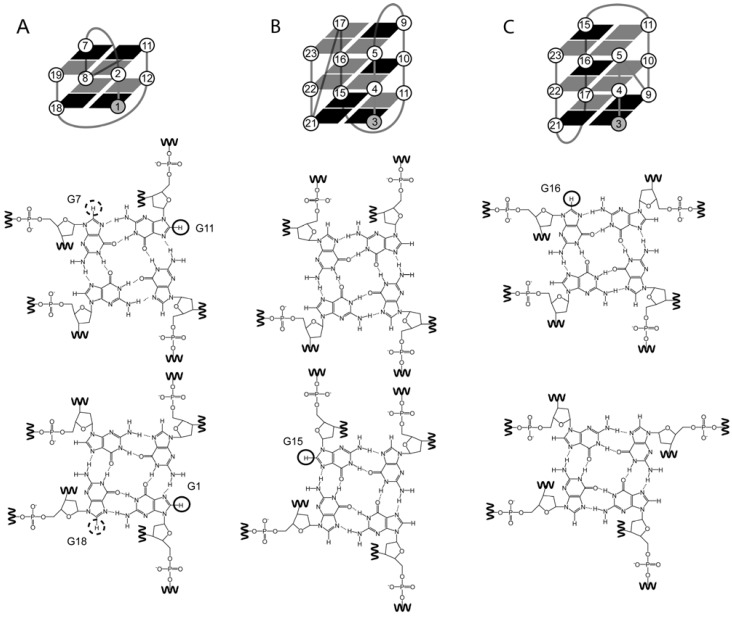
Schematic quadruplex topologies and their corresponding glycosidic bond angle combinations for tetrads is depicted below. The darker shaded areas in the topologies depict guanosines with *syn* glycosidic bond angle, and the lighter guanosines with *anti* glycosidic bond angle. The bases highlighted in the chemical structures depict the groove width combinations (medium, narrow, and wide) for the stacked tetrads indicate 8Br-Guanine C8 substitutions studied. The lower tetrad depicted corresponds to the 5'-end tetrad. In (**A**) the (d+pd) loop progression, in (**B**) the (-l-l-p) loop progression; and (**C**) the (-p-l-l) loop progression.

The d(G_2_T_4_G_2_CAG_3_T_4_G_2_T) sequence folds into a two-stacked quadruplex that adopts a topology defined by (d+pd) loop progression [7] (Figure 3A); This means that its first loop bridges a tetrad diagonally across- a diagonal loop. The second loop links the two-stacked tetrads within the same groove- a propeller loop. The progression of this loop is clockwise (+p). The third loop again bridges a tetrad diagonally across. Kuryavyi *et al.* substituted all eight bases of the quadruplex stem by 8Br-Guanine. Only the G(*syn*) substitutions G1 and G11 successfully folded into the same topology as the non-substituted sequence. The bromine atoms in both protrude into a wide groove and are thus sterically unhindered. Both G(*syn*) residues G18 and G7 protrude into a medium groove. Kuryavyi *et al.* could not define the topology to be the same as the unsubstituted one. But, they did not rule out that some of the topology might still exist as a mixture. The residues of a propeller loop may, or may not, interfere with a bromine atom protruding into the medium groove. Whilst in this study the bromine in a medium groove was destabilizing, the same is not true for a few other studies.

Phan *et al.*, utilized a series of 8Br-Guanine substitutions of the d(G_3_T_2_AG_3_T_2_AG_3_T_2_AG_3_T) human telomeric sequence to stabilize two different three-stacked topologies: loop progressions (-l-l-p) and (-p-l-l)- respectively Figure 3B,C [8]. This demonstrates that 8Br-Guanine can be utilized to influence the folding of a topology when the non-substituted sequence is in equilibrium. Substitution of G(*syn*) at position 15 drives the fold towards the (-l-l-p) topology. In this case the substitution has the bromine protruding into a medium groove. However, it appears in one of the outer tetrads of the quadruplex stem. Thus with less potential for steric hindrance. In the (-p-l-l) topology the modification appears with the bromine protruding into a wide groove and in the middle tetrad of the quadruplex stem; position 16. 

Identical structural features apply for the same topology studied by Matsugami *et al.* In this study they stabilized the d(AG_3_T_2_AG_3_T_2_AG_3_T_2_AG_3_) natural telomeric sequence in K^+^ by substituting all five guanines expected to be in *syn* conformation with 8Br-Guanine [9]. The bromine atom protrudes into the topology’s single wide groove for three of the substitutions- indeed, one for each of the three tetrads in the quadruplex stem. In the other two substitutions bromine protrudes into different medium grooves- both in one of the outer tetrads of the quadruplex stem. With any combination of four substitutions (instead of five) the same topology is achieved. 

**Figure 4:**
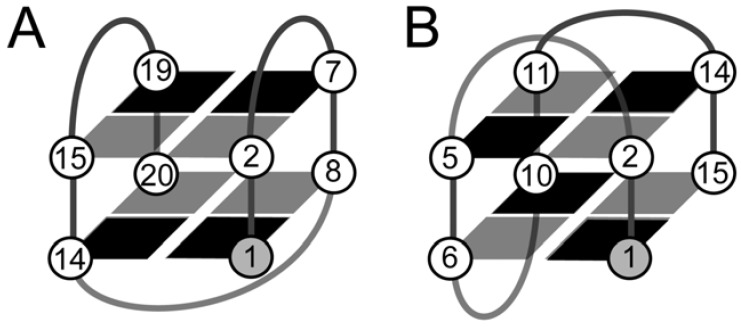
Schematic the two-stacked topologies defined by the (**A**) (-ld+l) loop progression; and (**B**) the (+l+l+l) loop progression. The dark shaded regions depict *syn* glycosidic bond angles and the light shaded *anti* glycosidic bond angles.

Lim *et al*. [10] utilized a G(*syn*) substitution on the human telomeric sequence d(G_3_T_2_AG_3_T_2_AG_3_T_2_AG_3_T) described above [8] to calculate the structure of the two-stacked (-ld+l) topology—Figure 4A [10]. In this case the structure was stabilized by a single G(*syn*) substitution with a bromine protruding into a wide groove. They unsuccessfully attempted substitutions that would have resulted in bromine atom protruding into the narrow groove.

In a systematic study of position C8 modifications the thermal stability of 8Br-Guanine substitutions on a (-p-l-l) topology formed by the human telomeric sequence d(T_2_G_3_T_2_AG_3_T_2_AG_3_T_2_AG_3_A) has been assessed [6]. The three substituents in wide grooves result in significant increase in thermal stability when compared to the unmodified sequence. A bromine in a medium groove crossed by a propeller loop results in a smaller increase in stability. However, a bromine in a medium groove not bridged by a propeller loop displays also a significant increase of stability comparable to that displayed by the bromines in wide grooves. This clearly indicates that it is the steric hindrance due to the presence of the loop in a medium groove that defines the stability of the quadruplex stem. 

It can be safely concluded that the magnitude of the steric hindrance in 8Br-Guanine substitutions prevents formation of narrow grooves, and for medium grooves the presence of propeller loop residues may destabilize the resulting topology.

### 4.2. The Effect of O-Methyl, Amino, and Methyl C8 Substitutions

The effect of any other known substitution is dominated by steric hindrance of the substituent; as with 8Br-Guanine. In general 8OMe-Guanine substitutions at a *syn* position have a stabilizing effect that is weaker than that of 8Br-Guanine, but higher than that of 8-aminoguanine [6]. The 8Me-Guanine substitution favors the *syn* GBA conformation similarly with 8Br-Guanine both in Z-DNA [11] and antiparallel quadruplexes [12,13]. 8Me-Guanine substitutions have been used in attempt to manipulate strand orientation [14]. A study in tetramolecular quadruplexes has shown that the 8-methylguanine modification may not always result in the *syn* GBA conformation [15]. The same study indicated that in some case this modification led to unusual quadruplex structures with all *syn* tetrads [15]. Randazzo’s laboratory has utilized 8Br-Guanine and 8Me-Guanine to demonstrate that these substituents provide steric hindrance to groove binding by ligands [16,17].

Substitution on a *syn* residue has produced a stabilizing effect in a quadruplex structure from the retinoblastoma susceptibility gene [18]. A similar stabilizing effect is implied by the increase in the thrombin inhibitory activity of a 15-mer quadruplex-forming oligodeoxynucleotide [13].

### 4.3. The Effect of 8Oxo-Guanine C8 Substitutions

Whilst in all other C8 modifications the purine dimensions are minimally modified, the 8Oxo-Guanine substitution results in a non-planar five-membered ring in the purine. Thus the substitution results in loss of the ability of this base to maintain its co-planarity with the rest of the bases in the tetrad [6].

### 4.4. Base Modifications in Thymine, Cytosine, and Adenine

The use of modified bases other than guanines has been devoted to aiding assignments, probing for pseudo-planar architectures, and shifting equilibrium towards single topology. The d(G_2_T_4_G_2_CAG_2_G T_4_G_2_T) sequence folds into a two-stacked quadruplex that adopts a topology defined by (d+pd) loop progression. In this study Kuryavyi *et al.* used a range of analogues (8Br-Guanine, 8Br-Adenine, dUracil, 5Br-dUracil, dInosine, 5Me-Cytosine and 2,6-diaminoPurine) to aid sequential assignments [7]. 5Me-Cytosine was used to aid the assignments of both aromatic (H6) and exchangeable protons (amino group at position 4). Thymines to dUracil and/or 5Br-dUracil substitutions resulted into chemical shift differences of the exchangeable and aromatic protons while they were especially useful in identifying a particular thymine residue by the loss of the methyl group resonances. 

In a similar study on the topology formed by the sequence d[A_2_G_2_T_4_A_2_G_2_]_2_ 8Br-Adenine was used to demonstrate that the perturbation of either adenine in a A(*syn*):A(*anti*) mismatch contributed to the prevention of quadruplex formation [19]. It would be expected that the A(*syn*) would not be perturbed since the bromine at position 8 would be favourable for this structural conformation. However, the perturbation can be accounted for by invoking inter-residue steric hindrance. Some evidence for this was derived for A(*syn*) in further substitutions. The authors substituted both adenines in the mismatch by the smaller 2,6-diaminoPurine and, whilst the A(*syn*) substitution allowed for folding of the architecture, the same was not true with the A(*anti*) substitution. In a similar case, 8Me-Adenine has been used to define the structural details of the tetramolecular assembly of AG_3_T and TAG_3_T. It encourages the *syn* configuration of adenine in the all-parallel topology [20].

Thymines to dUracil and/or 5Br-dUracil substitutions were also used to differentiate between two dimeric G-quadruplex conformers [21]. This demonstrates that their usefulness can be extended to shifting equilibrium towards a single conformation and/or stabilization of a particular topology as in the case of *Bombyx mori* telomeric sequence [22].

### 4.5. Abasic Sites

Quadruplexes can fold with loops consisting of abasic sites. These occur naturally and are the most frequent DNA lesion or a common intermediate in DNA repair mechanisms. Plavec’s group has studied the *same* dimeric four-stacked quadruplexes with diagonal loops consisting of 1',2'-dideoxyribose, propanediol, hexaethylene glycol, and thymine residues [23]. The quadruplex with non-nucleosidic loop residues fold faster, but are less stable than dimeric d(G4T4G4)_2_. This has been attributed to the absence of hydrogen-bonds, and absence of stacking between loop residues, among other interactions. In all parallel unimolecular quadruplexes with single residue loops, 1',2'-dideoxyribose loops provide increased thermal stability when compared to any other base [24]. A tetrahydrofuranyl analogue stabilizing abasic sites has been used to demonstrate stable tetramolecular quadruplex formation in single substitutions of the sequence d(TGGGGGT) [25]. Studies on the human telomeric sequence d[(G_3_T_2_A)_3_G_3_] showed that the formation of the three-stacked unimolecular (-ld+l) topology is not hindered by any of the 12 possible abasic site tetrahydrofuranyl substitutions, regardless of the observed reduction in thermodynamic stability [26]. A study on the d[TA(G_3_T_2_A)_3_G_3_] human telomeric sequence showed similar results, with preservation of the three stacked -(pll) topology for any of the 12 possible abasic site tetrahydrofuranyl substitutions, and with reduction in stability overall but especially for substitutions on the middle tetrad [27].

## 5. Ribo-Base Modifications in DNA and Vice-Versa

Riboguanosine (rG) substitutions can in principle be utilized for identification of G(*anti*) due to the fact that the C3'-endo preferred conformation of the sugar pucker favors this GBA. Tang and Shafer have made a series of rG modifications in the TBA sequence. The ones that proved successful in folding this topology were all in G(*anti*) positions [28]. The structure of a dimeric propeller-type RNA G-quadruplex formed by the human telomeric RNA sequence r(UAGGGUUAGGGU) has been determined in solution by NMR [29]. In this study all guanosine bases adopt their preferred anti conformation. They utilized site-specific deoxyribose substitutions to assist resonance assignments. The substituted residues are recognized thanks to their upfield-shifted H2′/H2′′ peaks.

## 6. Backbone Modifications

LNA are 2'-*O*-4'-C-methylene-linked ribonucleotide nucleic acid analogues [30,31] that result into a forced C3'-endo conformation which in turn favors the anti glycosidic bond angle configuration [32]. LNA is known to increase the affinity of oligonucleotides for DNA and RNA targets [33]. It is possible to influence the thermodynamically preferred structure of G-quadruplexes with the introduction of LNA modifications [34], increasing the possibility of obtaining parallel quadruplexes. In fact, NMR structures of tetramolecular quadruplexes with fully or partly LNA modified Guanine residues have resulted in all-parallel structures [35,36]. A unimolecular NMR G-quadruplex structure of the TBA with a single LNA modified guanosine at the 3'-end corresponding to a G(*anti*) retained its original topology [37]—Figure 4B. A systematic study with the *Oxytricha nova* telomeric sequence d[G_4_T_4_G_4_]_2_ in K^+^ (normally antiparallel) revealed that only one combination of four LNA modified Guanines gave rise to a well folded quadruplex where all eight guanines participate in the stem [38].

## 7. Inosine Studies

The guanosine to inosine substitution has been used in G-quadruplex structural studies for almost 20 years [39,40]. In the folded architectures the substituted residue still participates in the tetrad but causes chemical shift differences, and/or improved dispersion in the imino proton spectrum of the quadruplex. The only difference with guanosine in its chemical structure is the absence of the amino group which causes chemical shift differences mainly of its own imino proton but also of the aromatic proton of the hydrogen bonded guanosine in the tetrad. Numerous examples of single guanine-to-inosine substitutions resulting into significantly improved NMR spectra and/or a single quadruplex conformation exists [41,42,43,44,45,46,47].

## 8. Isotopic Enrichment

Partially or fully isotopically labelled nucleosides have been used in quadruplex NMR structural studies [7,48,49,50,51,52,53,54] to assist with resonance assignments. Isotope filtering techniques allow for the discrimination of intra- and intermolecular NOEs [55]. Such techniques, have been used in the structure determination of a symmetric dimeric quadruplex, allowing for the unambiguous differentiation of intra- and inter-strand contributions for topology-defining NOEs [56]. Although this approach is dependent on knowledge of the folding topology, other methods focus on the unambiguous discrimination between intra- and intermolecular hydrogen bonds in symmetric multimeric quadruplexes [57] without prior structural knowledge requirements. More details regarding methodological aspects of isotopic enrichment have been reviewed previously.

Of particular importance, is the site-specific low isotopic enrichment (1%–2%, ^15^N and/or ^13^C) as an aid in the sequential assignment of G-quadruplexes [58,59]. This cost-effective approach has become part of the established methodology for structural studies [21,60] of these molecules, resulting in unambiguous assignment of imino signals (^15^N) and or sugar protons (^13^C). This approach may become even less expensive by utilizing only ^15^N-enrichment. Site-specific 2% ^13^C, ^15^N-labeled samples can additionally be utilized to unambiguously assign methyl and H6 protons of Thymines, using ^13^C-filtered experiments [61].

Site-specific incorporation of ^15^N and ^13^C-labeled nucleoside (N^1^, N^2^, N^7^-^15^N, C^2^-^13^C-labeled guanine and N^1^, N^6^, N^7^-^15^N-labeled adenine) phosphonates have been used to resolve imino proton assignments of a guanine and adenine rich sequence forming a unique fold [19]. The structure determination was additionally aided by site-specific substitutions of 2,6-diaminoPurine and 8Br-Adenine for Adenine, 8Br-Guanine, 7-deazaGuanine and dInosine for Guanine, and dUracil and 5Br-dUracil for thymine [19], thus illustrating the full range of options in low isotopic enrichment and base modifications as a tool in quadruplex structure determination.

## 9. Perspectives

Currently, it is still too expensive to utilize near 100% isotopically labelled nucleic acids. In addition, in solution NMR structural studies it is customary to establish the predominance of a single fold before pursuing full structure determination. In this context the use of modified bases serves the purpose of selecting a topology from equilibria. Base modifications and substitutions are a convenient means of signal identification as well as probes for establishing pseudo-planar hydrogen bond alignments. All of these results improved spectra for which, in select cases, full structure determination has been performed. 

There are a number of modifications to nucleosides that improve the thermal stability of quadruplexes, or their resistance to enzymatic degradation. There are a number of modifications that have been used to manipulate the folding of quadruplexes. Thus one can expect many more structures determined for nucleoside-modified quadruplexes as therapeutic entities, for use as sensors, catalytic systems, or materials. Solution NMR structural studies will continue to be central to these applications. 
